# MiR-218-5p-dependent SOCS3 downregulation increases osteoblast differentiation inpostmenopausal osteoporosis

**DOI:** 10.1186/s13018-023-03580-4

**Published:** 2023-02-15

**Authors:** Qian Zhou, Lihua Zhou, Jun Li

**Affiliations:** 1 Department of Geriatrics, Huangshi Central Hospital, Hungshi, 435000 Hubei China; 2 Department of Orthopedics, Huangshi Central Hospital, No. 141 Tianjin Avenue, Huangshigang District, Hungshi, 435000 Hubei China

**Keywords:** SOCS3, miR-218-5p, Osteoporosis, Bone marrow stromal cells

## Abstract

**Background:**

Postmenopausal osteoporosis (POP) is a prevalent skeletal disease among elderly women. Previous study indicated that suppressor of cytokine signaling 3 (SOCS3) participates in the regulation of bone marrow stromal cell (BMSC) osteogenesis. Here, we further investigated the exact function and mechanism of SOCS3 in POP progression.

**Methods:**

BMSCs were isolated from Sprague–Dawley rats and treated with Dexamethasone (Dex). Alizarin Red staining and ALP activity assays were applied to assess the osteogenic differentiation of rat BMSCs under the indicated conditions. Osteogenic genes (ALP, OPN, OCN, COL1) mRNA levels were determined using quantitative RT-PCR. Luciferase reporter assay verified the interaction between SOCS3 and miR-218-5p. Rat models of POP were established in ovariectomized (OVX) rats to detect the in vivo effects of SOCS3 and miR-218-5p.

**Results:**

We found that silencing SOCS3 antagonized the suppressive effects of Dex on the osteogenic differentiation of BMSCs. SOCS3 was found to be targeted by miR-218-5p in BMSCs. The SOCS3 levels were negatively modulated by miR-218-5p in femurs of POP rats. MiR-218-5p upregulation promoted the BMSC osteogenic differentiation, while SOCS3 overexpression reversed the effects of miR-218-5p. Moreover, SOCS3 was highly expressed and miR-218-5p was downregulated in the OVX rat models, and silencing SOCS3 or overexpressing miR-218-5p alleviated POP in OVX rats by promoting osteogenesis.

**Conclusion:**

SOCS3 downregulation mediated by miR-218-5p increases osteoblast differentiation to alleviate POP.

## Introduction

Osteoporosis (OP) is a common systematic skeletal disorder with a high incidence rate [1]. Loss of bone and deterioration of bone tissue is main features of OP, which contribute to bone fragility and fracture [2]. Biochemical markers of bone turnover (BTMs), such as the bone alkaline phosphatase (bALP), procollagen type I N propeptide (PINP), serum cross-linked C-telopeptides of type I collagen (bCTx), and urinary cross-linked N-telopeptides of type I collagen (NTx), are used to manage therapy monitoring in osteoporotic patients [3, 4]. Dexamethasone (Dex), an immune inhibitor applied clinically for inflammations, immunological rejection, and autoimmune diseases. The administration of Dex may contribute to induction of OP [5, 6]. Compared to men, menopausal females are vulnerable to osteoporotic fractures [7]. Postmenopausal osteoporosis (POP) is a common type of OP [8]. It is a metabolic disorder resulted from decreased estrogen levels and declined ovarian function in postmenopausal women [9]. The basic pathogenesis of POP is involved an imbalance between bone formation by osteoblasts and bone resorption by osteoclasts. POP imposes a huge economic burden on postmenopausal women [10, 11]. Denosumab is an antiresorptive agent that reduces osteoclastogenesis and a widely known molecular-targeted drug for POP treatment [12, 13]. However, continuous treatment with denosumab reduces physiologic bone remodeling and causes potential adverse effects [14]. Study shows that the therapy for PMO should be personalized [15]. Thus, it is critical to investigate the mechanisms of POP to uncover effective biomarkers.

Bone marrow stromal cells (BMSCs) can differentiate into myoblasts, adipocytes, chondrocytes, and osteoblasts in vitro [16, 17]. The suppressor of cytokine signaling 3 (SOCS3) family is cytoplasmic adaptor protein that negatively modulates kinds of cytokine responses in leukocytes [18]. Upregulation of SOCS3 augments RANKL, TNF-α, and TGF-β-induced osteoclast formation via suppression of specific anti-osteoclastic JAK/STAT signaling pathways [19]. Elevated SOCS3 level is closely related to inflammation‑mediated bone loss [20]. Additionally, SOCS3 was found to suppress osteoblast differentiation via direct regulation of the phosphorylation of SMAD1 and STAT3 [21]. Therefore, SOCS3 plays a key role in bone biology. We speculated that SOCS3 affects bone formation by regulating osteogenic differentiation of BMSCs.

Recent evidence has highlighted on the role of noncoding RNAs in musculoskeletal conditions, such as tendon injuries, osteoarthritis, and rheumatoid arthritis [22–25]. MicroRNAs (miRs) are single-stranded, short noncoding RNAs with about 22 nucleotides, playing important roles in posttranscriptional gene regulation. [26, 27]. Increasing studies have revealed that miRs can regulate osteogenic differentiation in the pathological processes OP [28, 29]. MiR-21-5p inhibits osteoclast differentiation in vitro and suppresses bone resorption in vivo by downregulating SKP2 in OP [30]. MiR-151b expression is elevated in the bone of OP rats, and miR-151b suppresses osteoblast proliferation, differentiation, and mineralization by targeting Msx2 [31]. These literatures suggest that the investigations on the function and mechanism of osteogenesis-associated miRs may be helpful to develop possible strategies for bone formation.

Here, we aimed to explore the function and molecular mechanism of SOCS3 mediated by miR in the progression of OP in vitro and in vivo. The findings of our study may provide clues for OP treatment.

## Materials and methods

### Animal experiments

All procedures in animal experiments were performed following the NIH Guides for the Care and Use of Laboratory Animals and were approved by the Ethics Committee of Huangshi Central Hospital. Eighty female Sprague–Dawley rats (3 months, 105–145 g) were provided by Beijing Vital River Laboratory Animal Technology (Beijing, China). In one experiment, the rats were divided into four groups: sham + AAV-sh-NC, sham + AAV-sh-SOCS3, OVX + AAV-sh-NC, OVX + AAV-sh-SOCS3. In another experiment, the rats were divided into sham group, OVX group, OVX + AAV-vector group, and OVX + AAV-miR-218-5p group. *N* = 10 in each group. The rats in the sham or OVX group were first anesthetized with 1.5% isoflurane in oxygen and then experienced either sham operation (back incision but not removed bilateral ovaries) or bilateral ovariectomy (bilateral ovary removal). The adeno-associated virus (AAV) serotype 9 carrying sh-SOCS3 were designed by Genechem (Shanghai, China). Six weeks after the surgery, the rats were i.v. injected with 200 μl AAV-sh-SOCS3, AAV-sh-NC, AAV-vector, or AAV-miR-218-5p (8 × 10^11^ genome copies/rat). Seven weeks after injection, the rats were sacrificed by cervical dislocation and the distal femurs were separated.

### BMSC isolation and treatment

BMSCs in the femurs were collected from both sham and OVX rats as previously described [32]. Briefly, the bilateral lower limb femurs were collected under aseptic conditions and were stored in a culture dish. DMEM (5 ml; Sigma-Aldrich, USA) containing 10% FBS (Sigma-Aldrich) were mixed with 0.5 ml heparin. Then, this mixed solution was used to wash the marrow cavities of the femurs several times. The flushing fluid was shaken fully and resuspended in DMEM containing 10% FBS. The cells at the density of 1 × 10^6^ cells/ml were added to a culture bottle (25 cm^2^) and incubated at 37 °C with 5% CO_2_. When the cells reached 80%-90% confluence, fresh α-MEM was used for cell culture for 24 h. To induce osteogenic differentiation, rat BMSCs were incubated with osteogenic medium. For Dex treatment, 4 uM Dex was added to culture with the cells.

### Cell transfection

MiR-218-5p inhibitor and mimics were designed by Guangzhou RiboBio Co., Ltd. For SOCS3 overexpression, pcDNA3.1/SOCS3 was constructed by GenePharma (Shanghai, China) and pcDNA3.1 empty vector served as internal control. The short hairpin RNA (sh-SOCS3) and a scrambled shRNA (sh-NC) were purchased from Shanghai GeneChem Co., Ltd, which were inserted into the pGPH1/Neo vector (Axel Biotechnology Inc.). The constructed pGPH1/Neo vector was transfected into BMSCs. Lipofectamine 2000 (Invitrogen, USA) was used to perform the transfection into rat BMSCs for 48 h.

### RT-qPCR

TRIzol reagent (Takara Biotechnology, Dalian, China) was used to extract total RNA in BMSCs. Then the RNA was reverse transcribed into cDNA using PrimeScript RT reagent kit (Takara). The qPCR was performed in ABI 7500HT real‑time PCR system (Thermo Fisher Scientific) by using SYBR Premix EX Taq Kit (Takara). The relative RNA expression of miR-218-5p, SOCS3, ALP, OPN, OCN, COL1 was calculated using the 2^−∆∆Ct^ method normalized to GAPDH and U6.

### Alizarin Red staining and ALP activity

After indicated transfection or Dex treatment, rat BMSCs were seeded in 6-well plates (1 × 10^6^ per well) and fixed with 4% paraformaldehyde for 10 min. Then the BMSCs were stained with 40 mM Alizarin Red for 20 min at room temperature. The BMSCs were lysed in lysis buffer containing 0.1% Triton X-100. ALP activity of the cell supernatants was detected using ALP colorimetric assay kit (BioVision, USA) at 405 nm under a micro-plate reader.

### Western blotting

RIPA buffer (Beyotime, Shanghai, China) was used to extract total protein in BMSCs. The protein concentration was assessed by a BCA Protein Assay Kit. Then the collected proteins (40 μg) were isolated with electrophoresis on 10% SDS-PAGE and transferred onto PVDF membranes. Subsequently, the membranes were blocked with 5% non-fat milk and then cultured with primary antibody of anti-SOCS3 (#ab280884, 1/1000, Abcam) overnight at 4 °C. Next, the membranes were further incubated with the secondary antibodies in the dark for 2 h at room temperature. The protein bands were measured by a chemiluminescence detection system (Amersham, Little Chalfont, UK).

### H&E staining

Rat femurs were fixed overnight with 4% paraformaldehyde (Sigma-Aldrich). After decalcification for 14 days in 10% EDTA (Solarbio, Beijing), the femurs were paraffin-embedded and cut into 5-μm sections. Then, the femur sections were stained with hematoxylin and eosin (H&E) to observe bone trabecular area under a microscope (Olympus BX53).

### Luciferase reporter assay

The SOCS3 3′UTR was inserted into the pGL3 vector to construct SOCS3-Wt and SOCS3-Mut was generated by site-directed mutagenesis. SOCS3-Wt and SOCS3-Mut were co-transfected with the miR-218-5p inhibitor and NC inhibitor into BMSCs using Lipofectamine 2000. After 48 h, the luciferase activity of SOCS3-Wt and SOCS3-Mut was measured using Dual-Luciferase Reporter Assay System (Promega).

### Statistical analysis

Data are expressed as mean ± SD and were analyzed using SPSS 18.0 software (SPSS Inc., Chicago, USA). All experiments were performed three times, independently. The normal distribution of the data was tested using the Shapiro–Wilk test. Student’s t-test or one-way ANOVA was utilized to analyze the differences of groups, with *p* value < 0.05 being statistically significant.

## Results

### SOCS3 silencing promotes BMSC osteogenesis

**Fig. 1:**
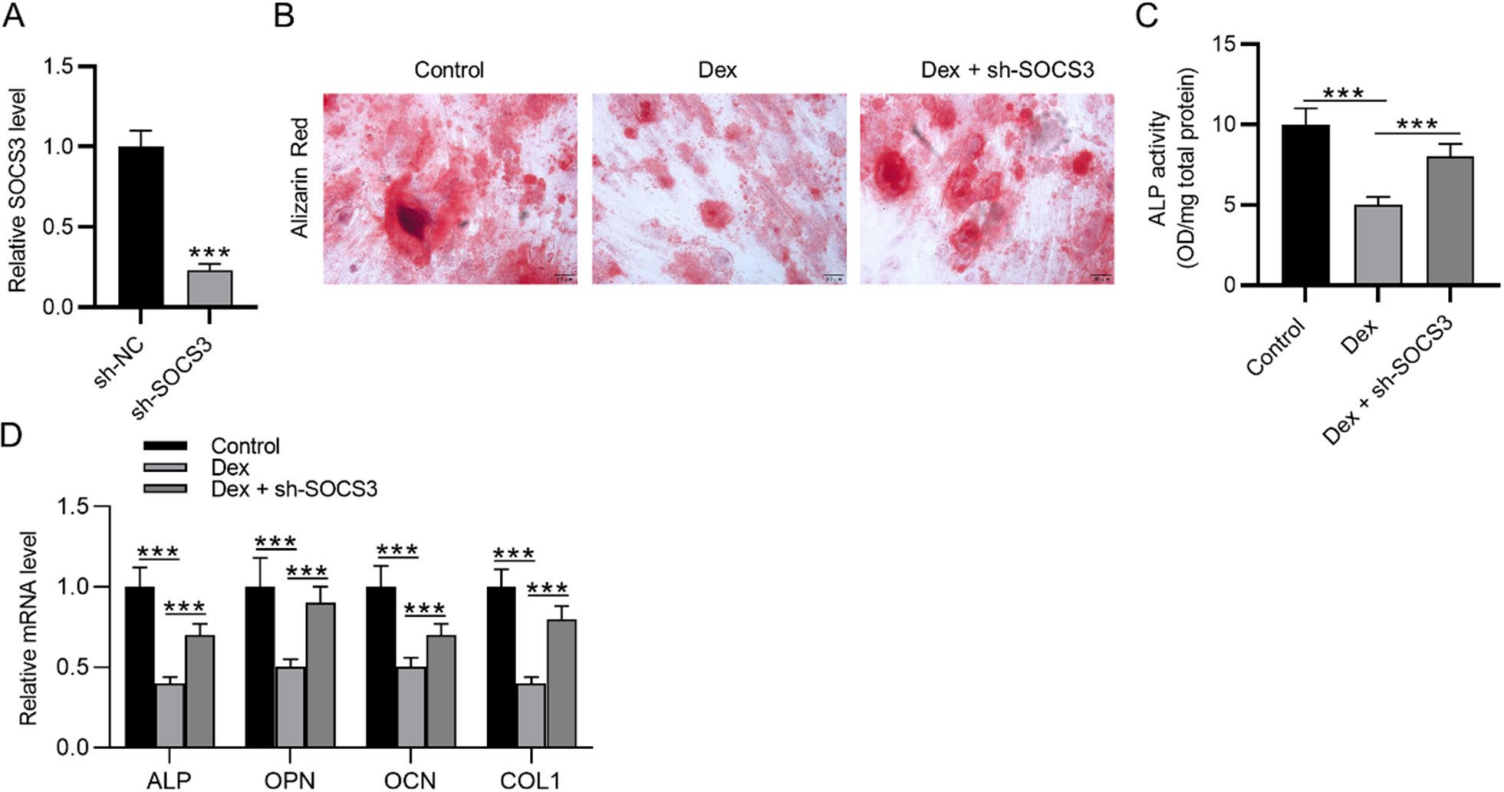
SOCS3 silencing promotes BMSC osteogenesis. **A** The SOCS3 level in rat BMSCs after transfection of sh-SOCS3 was detected by RT-qPCR. **B** Alizarin red staining was used to evaluate the osteogenesis efficiency of BMSCs after Dex treatment or Dex treatment in combination with sh-SOCS3. **C** ALP activity was measured in the control, Dex, and Dex + sh-SOCS3 groups. **D** RT-qPCR was used to detect the mRNA expression of osteogenesis markers (ALP, OPN, OCN, COL1) in BMSCs after the indicated treatments. ***p < 0.001

Dex could suppress BMSC osteogenesis. We investigated whether SOCS3 regulates BMSC osteogenesis under Dex treatment. The silencing efficiency of SOCS3 in rat BMSCs was verified using RT-qPCR (Fig. 1A). The transfected BMSCs were treated with Dex. Alizarin red staining was used to evaluate the osteogenic differentiation of BMSCs. Dex treatment decreased BMSC osteogenic differentiation compared to control, while SOCS3 downregulation significantly elevated calcium accumulation in BMSCs and reversed the Dex-induced inhibition of BMSC osteogenesis (Fig. 1B). The ALP activity was revealed to be decreased in the Dex group, and silencing SOCS3 elevated the ALP activity in BMSCs with Dex treatment (Fig. 1C). The mRNA expression of osteogenesis markers (ALP, OPN, OCN, COL1) was detected using RT-qPCR. We found that ALP, OPN, OCN, COL1 mRNA levels were reduced after Dex treatment, which was rescued by SOCS3 downregulation (Fig. 1D). Therefore, silencing SOCS3 could antagonize Dex and promote BMSC osteogenesis.

### SOCS3 is targeted by miR-218-5p

**Fig. 2:**
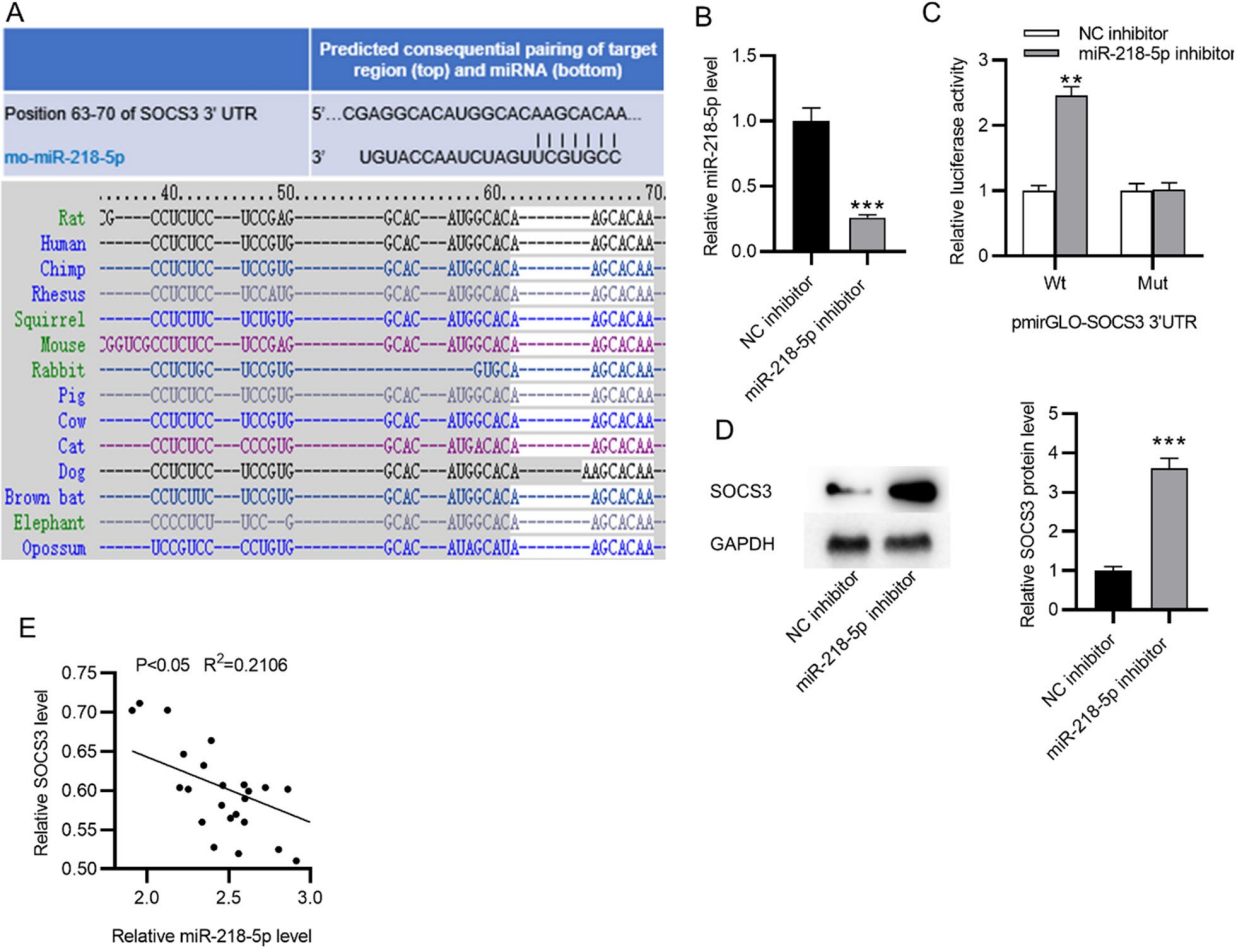
SOCS3 is targeted by miR-218-5p. **A** The potential binding site of miR-218-5p at position 63–70 of SOCS3 3′UTR. **B** The miR-218-5p level in BMSCs after transfection of miR-218-5p inhibitor. **C** The luciferase reporter assay in BMSCs post-transfection of miR-218-5p inhibitor and the indicated plasmids. **D** The protein level of SOCS3 in BMSCs after miR-218-5p silencing was measured using western blotting. **E** Pearson correlation analysis of the expression relationship between miR-218-5p and SOCS3 in the bone tissues of OVX rats. **p < 0.01; ***p < 0.001

We then explored the regulatory mechanism of SOCS3. Eight miRs (miR-218-5p, miR-455-5p, miR-124-3p, miR-203-3p, miR-384-5p, miR-22-3p, miR-19-3p, miR-30-5p) were predicted to possess binding site for SOCS3 among vertebrates at the Targetscan website (http://www.targetscan.org/). Among these miRs, miR-218-5p was reported to alleviate POP by contributing to the osteoblast differentiation of BMSCs [33]. Therefore, we decided to explore whether there is a relationship between SOCS3 and miR-218-5p in POP. The potential binding site of miR-218-5p at position 63–70 of SOCS3 3′UTR was shown to be conserved in species (Fig. 2A). Then we demonstrated the silencing efficiency of miR-218-5p inhibitor in BMSCs (Fig. 2B). The luciferase reporter activity of SOCS3-Wt was significantly elevated after miR-218-5p silencing, while that of SOCS3-Mut showed no significant change (Fig. 2C), suggesting the targeted relationship between them. Then, we assessed the effect of miR-218-5p knockdown on SOCS3 expression in BMSCs. Western blotting showed that miR-218-5p silencing elevated the protein expression of SOCS3 in BMSCs (Fig. 2D). According to Pearson correlation analysis, the expression of miR-218-5p and SOCS3 was negatively correlated in the bone tissues of OVX rats (Fig. 2E).

### MiR-218-5p facilitates osteogenic differentiation of BMSCs by targeting SOCS3

**Fig. 3:**
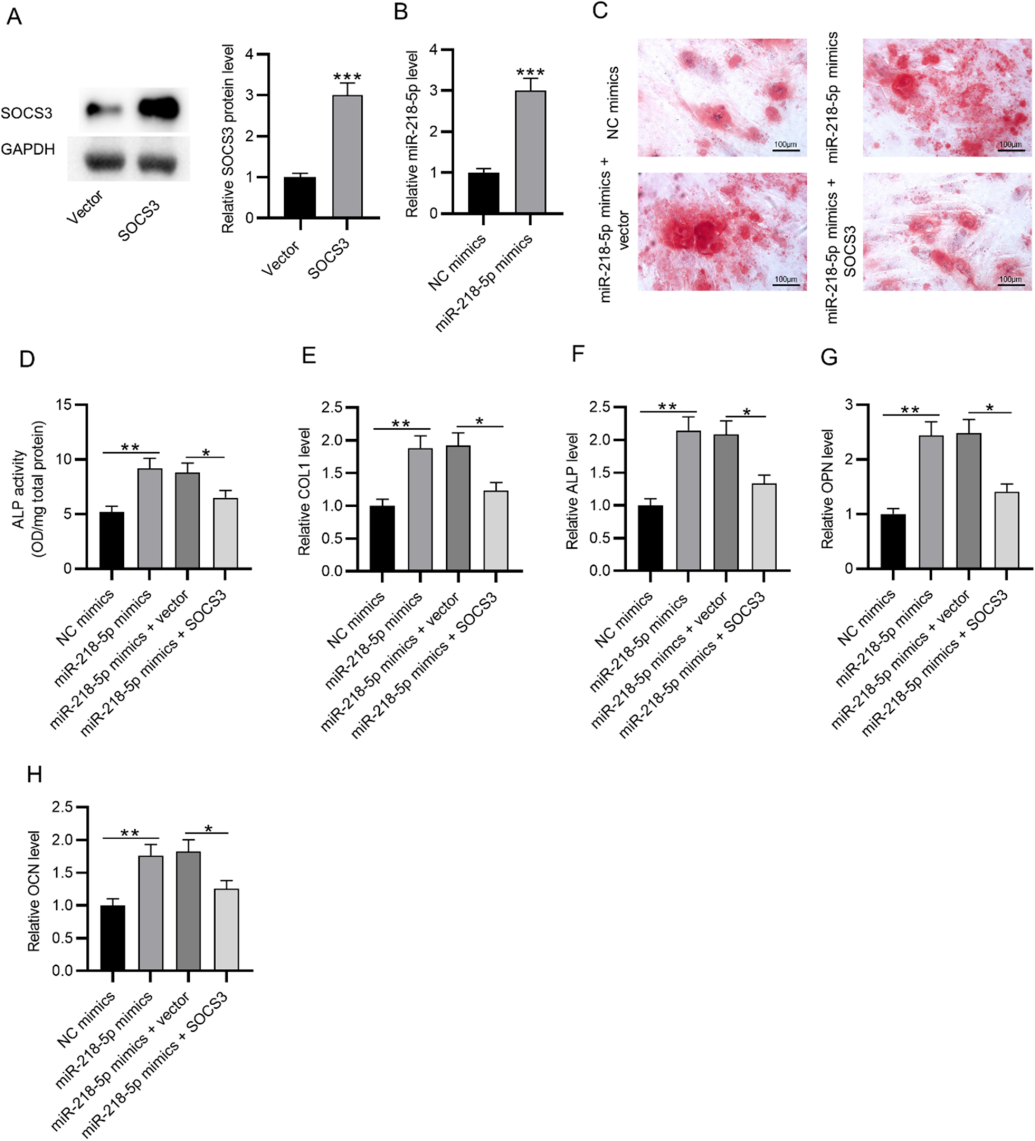
MiR-218-5p facilitates osteogenic differentiation of BMSCs by targeting SOCS3. **A** SOCS3 protein expression in BMSCs post-transfection of SOCS3. **B** MiR-218-5p expression in BMSCs post-transfection of miR-218-5p mimics. **C** Alizarin red staining was used to assess the osteogenic differentiation of BMSCs after miR-218-5p overexpression alone or in combination with SOCS3. **D** ALP activity in BMSCs after the indicated transfection. **E**–**H** The mRNA expression of osteogenesis markers (ALP, OPN, OCN, COL1) in BMSCs transfected with NC mimics, miR-218-5p mimics, miR-218-5p mimics + vector, and miR-218-5p mimics + SOCS3. *p < 0.05; **p < 0.01; ***p < 0.001

The SOCS3 protein level was markedly upregulated after the transfection of SOCS3 in BMSCs (Fig. 3A). The miR-218-5p mimics also elevated miR-218-5p expression (Fig. 3B). According to Alizarin red staining, SOCS3 upregulation reversed the promotive effect of miR-218-5p overexpression on osteogenic differentiation of BMSCs (Fig. 3C). Moreover, SOCS3 overexpression reversed the increase in ALP activity induced by miR-218-5p overexpression in BMSCs (Fig. 3D). The mRNA expression of osteogenesis markers (ALP, OPN, OCN, COL1) was elevated in BMSCs after the transfection of miR-218-5p mimics, which was antagonized by SOCS3 upregulation (Fig. 3E–H).

### SOCS3 silencing inhibits POP progression in vivo

**Fig. 4:**
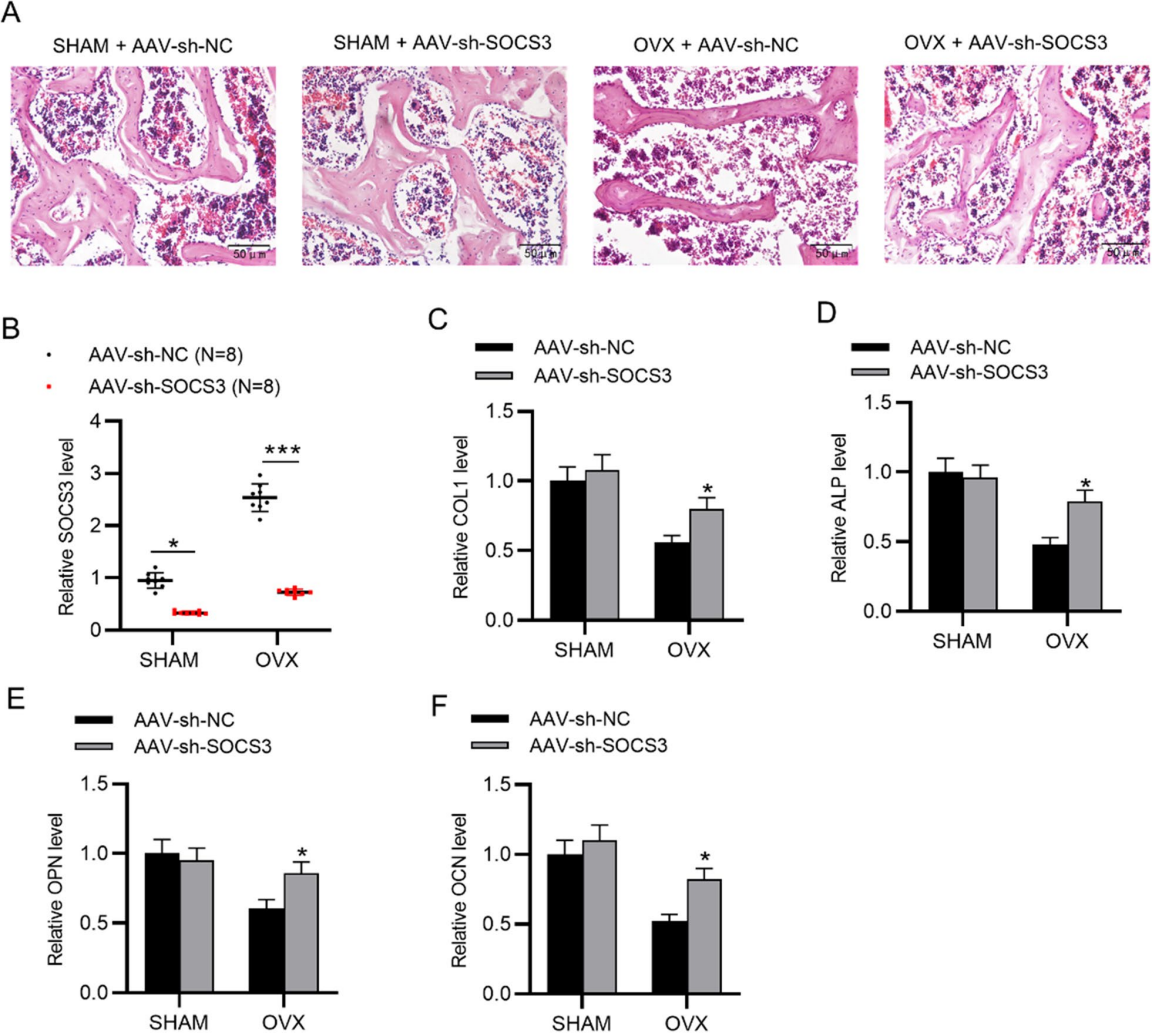
SOCS3 silencing inhibits POP progression in vivo. **A** H&E staining was used to assess the morphological changes of femurs in the sham or OVX rats after the indicated administration. **B** SOCS3 expression in OVX rat bone tissues after the administration of AAV-sh-SOCS3 was assessed by RT-qPCR. **C**–**F** The mRNA levels of osteogenesis markers (ALP, OPN, OCN, COL1) in bone tissues of sham or OVX rats after indicated treatments. *p < 0.05; ***p < 0.001

According to H&E staining of the rat femurs, the OVX group presented irregular and discontinuous trabecular bone compared to the sham group, and the administration of AAV-sh-SOCS3 significantly restored the morphological changes of OVX rat bone tissues (Fig. 4A). RT-qPCR analysis indicated that SOCS3 was upregulated in OVX rat bone tissues, and the administration of AAV-sh-SOCS3 significantly decreased the expression of SOCS3 (Fig. 4B). The reduction in mRNA levels of osteogenesis markers (ALP, OPN, OCN, COL1) in OVX rat models was reversed by AAV-sh-SOCS3 (Fig. 4C–F).

### Overexpression of miR-218-5p promotes bone formation in POP mice

**Fig. 5:**
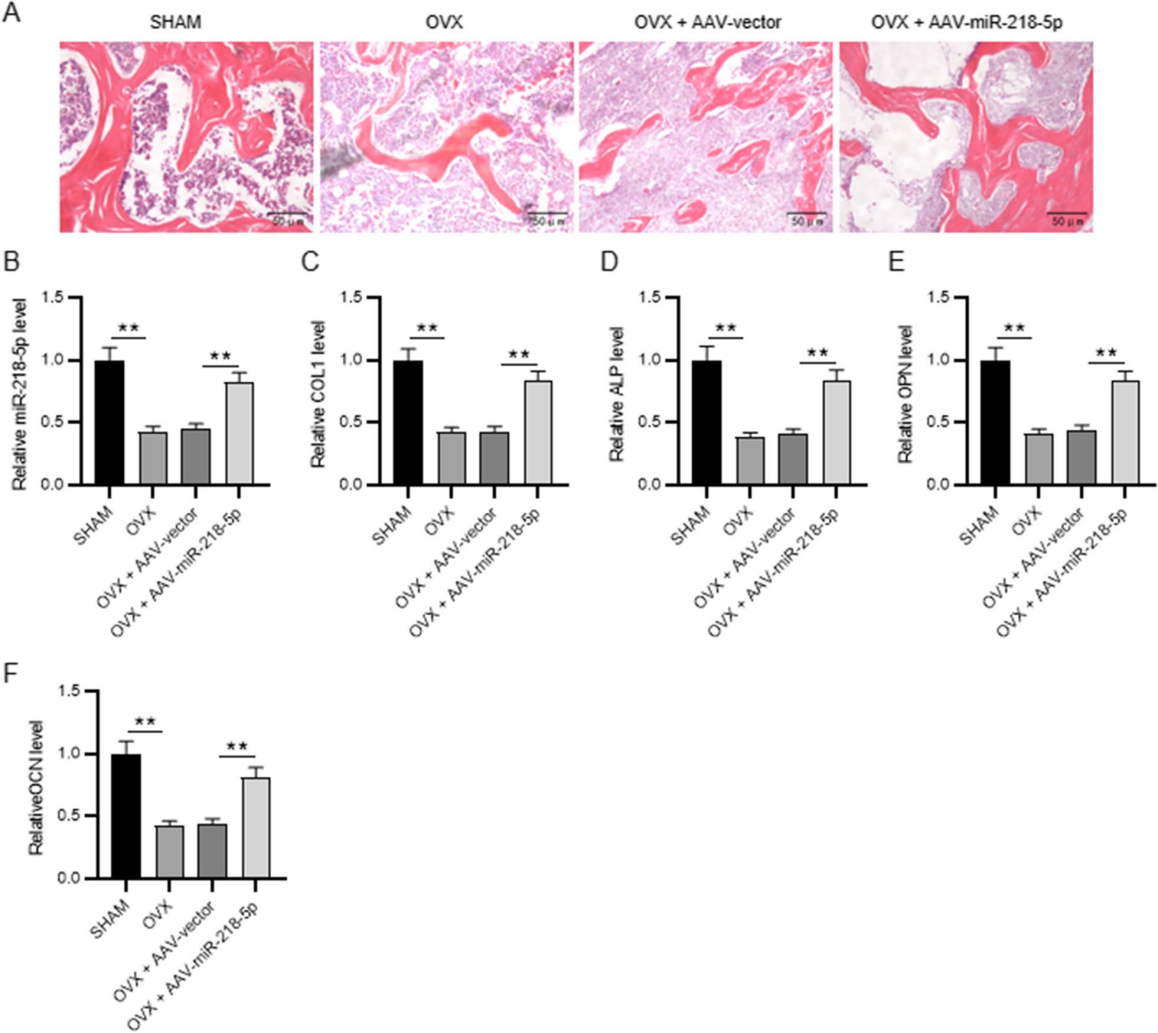
Overexpression of miR-218-5p promotes bone formation in POP mice. **A** H&E staining was used to assess the morphological changes of rat femurs after the indicated administration. **B** The miR-218-5p expression in OVX rat femurs after the administration of AAV-miR-218-5p was assessed by RT-qPCR. **C**–**F** The mRNA levels of osteogenesis markers (ALP, OPN, OCN, COL1) in the bone tissues of sham or OVX rats after indicated treatments. **p < 0.01

To further support our conclusion, the effect of miR-218-5p on POP in vivo was verified in our animal models. H&E staining of the rat femurs showed that the structure of bone trabeculae was relatively complete, and bone was obviously preserved in the sham group. In the OVX and OVX + AAV-vector groups, a large quantity of bone trabeculae was lost, and the bone loss was obvious. Bone loss in the bone trabeculae was alleviated, and most of its structure was preserved in the OVX + AAV-miR-218-5p group (Fig. 5A). Additionally, reduced mRNA levels of miR-218-5p, COL1, ALP, OPN, and OCN in the OVX and OVX + AAV-vector groups were significantly restored in the OVX + AAV-miR-218-5p group (Fig. 5B–F).

## Discussion

BMSCs from OP patients have dramatically poorer abilities to differentiate into osteoblasts than into adipocytes, which contributes to bone loss [34]. According to previous research, enhanced SOCS3 protein stability in osteoblasts and BMSCs is crucial for CUE domain-containing 2-mediated suppression of osteoblast differentiation [35]. SOCS3 silencing reverses the suppressive effects of leukemia inhibitory factor (LIF) on osteoblast differentiation of BMSCs [36]. Here, we used Dex to treat BMSCs, which caused the suppression of osteogenic differentiation of BMSCs. We found that silencing SOCS3 could counteract Dex and promote BMSC osteogenesis by augmenting ALP activity and upregulating osteogenesis markers (ALP, OPN, OCN, COL1) levels. These findings suggested that suppression of SOCS3 may rescue decreased abilities of BMSCs to differentiate into osteoblasts. OVX promotes bone resorption and inhibits bone formation, which is similar to POP-like characteristics [37]. Therefore, OVX has been widely applied for establishment of animal models of POP [38]. In this study, we used OVX rat models to detect the in vivo effects of SOCS3. We found that SOCS3 was upregulated in OVX rat models. The administration of AAV-sh-SOCS3 restored the morphological changes of OVX rat bone tissues and upregulated levels of osteogenesis markers. These results suggested that suppression of SOCS3 rescued bone loss in OVX rats by promoting osteogenesis. However, a previous report indicated that SOCS3 is downregulated in blood samples of POP patients, which is contradictory to our finding. Therefore, it is needed to increase sample size or apply multiple models to obtain more convincing conclusion.

MiRs are reported to play multifaceted roles in regulating cellular functions by inhibiting target genes [39, 40]. In physiological processes, miRs regulate bone formation and bone resorption, thus contributing to the maintenance of bone homeostasis. Under pathological conditions, a deregulation of miR signaling contributes to the onset and progression of skeletal disorders, e.g. POP [41]. MiRs can regulate different aspects of BMSCs such as adipogenesis and osteogenesis [42]. MiR-218 is able to induce osteoblast differentiation by targeting different inhibitors of the Wnt pathway, such as SFRP2, DKK2, TOB1, SOST, DKK2 [43]. Furthermore, this miRNA is also implicated in the inhibition of osteoclast differentiation by targeting TLR4 mRNA in macrophages [44]. Kou et al. indicated that miR-218-5p relieves POP by facilitating osteoblast differentiation of BMSCs [33]. However, the exact mechanisms through which miR-218-5p regulates the bone remodeling under POP conditions, are still uncharacterized. Moreover, the regulatory role of miR‐218‐5p on the bone formation, as well as the osteoblast phenotype in POP models has not been evaluated. Here, miR-218-5p was revealed to bind to the SOCS3 3′UTR. Moreover, the SOCS3 level was negatively modulated by miR-218-5p. We further found that miR-218-5p facilitated BMSC osteogenic differentiation by upregulating osteogenesis marker levels in BMSCs. Interestingly, SOCS3 overexpression counteracted the effects of miR-218-5p on BMSC osteogenic differentiation in vitro. This suggested the interaction between miR-218-5p and SOCS3 in regulating BMSC osteogenic differentiation. To further support our conclusion, the effect of miR-218-5p on bone formation in POP rat models was verified in this study. Our results showed that injection of AAV-miR-218-5p significantly preserved bone trabeculae and rescued bone loss in OVX rats. Additionally, the levels of osteogeneis-related markers were restored by miR-218-5p overexpression. The in vivo experiments further confirmed that miR-218-5p alleviated POP by increasing osteoblast differentiation via suppression of SOCS3.

In conclusion, our findings support the hypothesis that SOCS3 downregulation mediated by miR-218-5p alleviates POP-like symptoms by promoting osteogenesis. This study suggests that application of inhibiting SOCS3 to the bone marrow microenvironment may provide a potential therapeutic strategy for POP-related bone loss.

## Data Availability

The datasets used during the current study are available from the corresponding author on reasonable request.
